# Microwave Assisted Enzymatic Kinetic Resolution of (±)-1-Phenyl-2-propyn-1-ol in Nonaqueous Media

**DOI:** 10.1155/2014/482678

**Published:** 2014-02-23

**Authors:** Saravanan Devendran, Ganapati D. Yadav

**Affiliations:** Department of Chemical Engineering, Institute of Chemical Technology, Matunga, Mumbai 400 019, India

## Abstract

Kinetic resolution of 1-phenyl-2-propyn-1-ol, an important chiral synthon, was studied through trans-esterification with acyl acetate to investigate synergism between microwave irradiation and enzyme catalysis. Lipases from different microbial origins were employed for the kinetic resolution of (R/S)-1-phenyl-2-propyn-1-ol, among which *Candida antarctica* lipase B, immobilized on acrylic resin (Novozym 435), was found to be the best catalyst in *n*-hexane as solvent. Vinyl acetate was the most effective among different acyl esters studied. The effect of various parameters was studied in a systematic manner. Definite synergism between microwave and enzyme was observed. The initial rate was improved around 1.28 times under microwave irradiation than conventional heating. Under optimum conditions, maximum conversion (48.78%) and high enantiomeric excess (93.25%) were obtained in 2 h. From modeling studies, it is concluded that the reaction follows the Ping-Pong bi-bi mechanism with dead end alcohol inhibition. Kinetic parameters were obtained by using nonlinear regression. This process is green, clean, and easily scalable as compared to the chemical process.

## 1. Introduction

Enantiomeric pure chemicals are needed for synthesis of optically active compounds that have significant market values across a variety of industries [1], among which chiral secondary alcohols have several applications in pharmaceutical and chemical industries, such as chiral auxiliaries, and they can be easily derivatized with different functional groups [2]. In comparison with classical chemical methods such as preferential crystallization, diastereomerization, chromatographic separation, and asymmetric reduction by chiral metal oxides, biocatalytic processes are broadly accepted as good options to prepare optically pure secondary alcohols [3]. Biocatalysts score over chemical processes because they often possess high stereo-, chemo-, and regio-selectivity. Biocatalytic systems can be operated at mild operating conditions that reduce byproduct formations and are recyclable and easily adoptable in nonaqueous and neoteric solvents. Biocatalytic processes are reported as simple and cost effective for the synthesis of single enantiomeric compounds. However, the major drawback of these processes is the fact that they are slow in nature and need to be intensified in order to meet industrial requirements [4–6].

Among various biocatalytic paths, lipase catalyzed kinetic resolution of racemic mixtures have been favored for preparing the optically active secondary alcohols through hydrolysis and transesterification reactions. Lipases have inherent ability to accept a broad range of substances and do not require expensive cofactors like NAD(P)H. Lipases are readily available from animal, plant, and microbial sources and remain active in both organic and neoteric solvents like ionic liquids and supercritical carbon dioxide (scCO_2_). However, alcohol dehydrogenases catalyzed asymmetric reduction requires expensive cofactors for its catalytic activity and is also less adoptable to nonaqueous media [7, 8].

In recent years, microwave irradiation, a green and clean alternative energy source, has been employed to enhance the reaction rates and selectivities for organic synthesis as well as materials production [9, 10]. In the conventional heating, the rate of heat transfer from external heating source to reaction system depends on the thermal conductivity of reaction vessel, which might lead to higher temperature at vessel surface than the reaction mixture. Hence, it requires longer equilibrium time and is difficult to control. On the contrary, microwave irradiation results in efficient internal heating by direct coupling of microwave energy with polar molecules such as solvents, reagents, or catalysts in the vessel. Microwaves pass through the wall of reactor and are converted into heat directly in bulk mass of material in the vessel. It is irrespective of the thermal conductivity of vessel material. The temperature gradient observed in microwave heating is exactly opposite to that in conventional heating. Thus, it reduces the reaction time [11]. The synergism with microwave irradiation is found to enhance the reaction rate in enzymatic reactions [12]. The rate of lipase catalyzed reaction is enhanced to several folds as compared to conventional heating and the lipase is quite stable under microwave irradiation [13, 14].

**Scheme 1:**
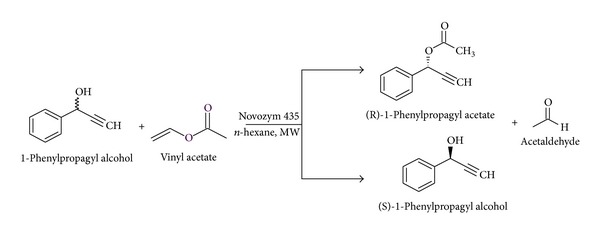
Lipase catalysed transesterification of (R/S)-1-phenylpropagyl alcohol with vinyl acetate under microwave irradiation.

Optically active 1-phenyl-2-propyn-1-o1 and its substituted derivatives are important precursors for synthesis of enantiomeric pure natural compounds and biologically active molecules such as eicosanoids and macrolides antibiotics [15]. They can also be used as starting materials for stereoselective synthesis of polyhydroxylated compounds, allenes, benzo[*b*]furan (1-benzofuran), and derivatives [16, 17]. There are a few studies reported on the synthesis of the single enantiomer of 1-phenyl-2-propyn-1-o1 by asymmetric hydrolysis by using microbes, asymmetric chemical catalysis, or lipase catalyzed kinetic resolution under conventional heating [18–23]. All these processes require either longer reaction time or high enzyme loading and there is no information on kinetics of the reaction, which is required for reactor design and scale-up. In the current work, microwave assisted lipase catalyzed transesterification of (R/S)-1-phenyl-2-propyn-1-o1 was undertaken with vinyl acetate in nonaqueous media (Scheme 1). The effect of various parameters such as different commercially available immobilized lipases, acyl donors, solvents, agitation speed, temperature, catalyst loading, and acyl donor concentration was studied systematically by varying one parameter at a time. Finally, the mechanism was proposed and kinetics were developed. All results are novel and useful to scale up this process.

## 2. Materials and Methods

### 2.1. Enzymes

The following enzymes were received as gift samples from M/s Novozymes A/S (Bagsvaerd, Denmark): (i) Novozym 435: Lipase B from *Candida antarctica*, supported on a macroporous acrylic resin with a water content of 1-2% (w/w) and enzyme activity 10,000 PLU/g, (ii) Lipozyme RM-IM: Lipase from *Rhizomucor miehei*, supported on a macroporous anion exchange resin with a water content of 2-3% (w/w) and enzyme activity 6 BAU/g, and (iii) Lipozyme TL IM: Lipase from *Thermomyces lanuginosus*, supported on porous silica granulates with water content 1-2% and enzyme activity 175 IU/g.

### 2.2. Chemicals

Hexane, toluene, cyclohexane, isopropyl ether, vinyl acetate, and other analytical and HPLC grade reagents were purchased from M/s S.D. Fine Chemicals Pvt. Ltd., Mumbai, India. (±)-1-Phenyl-2-propyn-1-ol, vinyl butyrate and vinyl laurate were purchased from Sigma-Aldrich India Pvt. Ltd., Bangalore, India. All chemicals and enzymes were used without any further modification/purification.

### 2.3. General Experimental Setup 

#### 2.3.1. Conventional Heating

The experimental setup consisted of a 3 cm i.d. mechanically agitated glass reactor of 50 cm^3^ capacity, equipped with four baffles and a six-bladed turbine impeller. The entire reactor assembly was immersed in a thermostatic water bath, which was maintained at a predetermined temperature with an accuracy of ±1°C. A typical reaction mixture consisted of 0.001 mol racemic alcohol and 0.001 mol vinyl ester diluted to 15 cm^3^ with *n*-hexane as a solvent. The reaction mass was agitated at 60°C for 15 min at a speed of 300 rpm and then 10 mg of enzyme was added to initiate the reaction.

#### 2.3.2. Microwave Heating

The studies were carried out in a commercial microwave reactor assembly (Discover, CEM-SP1245 model, CEM Corp., Matthews, NC, USA) at the desired temperature. The reactor was a 100 mL capacity, 4.5 cm i.d. cylindrical glass vessel with a provision for mechanical stirring. A standard six-blade-pitched turbine impeller of 1.5 cm diameter was used for agitation. However, the actual reactor volume exposed to the microwave irradiation was 45 mL with 5.5 cm height. The temperature in the reactor was computer controlled. The quantities of reactant and enzyme used for microwave reaction procedure were identical to those used for conventional heating.

### 2.4. Analysis

Reaction progress and enantiomeric excess (ee) were monitored by periodic withdrawal of clear liquid samples from the reaction mixture which were analyzed by high performance liquid chromatography (HPLC) (1260 infinity series, Agilent technologies, CA, USA) equipped with chiralpak- IB analytical column (250 × 4.6 mm ID) (Daicel Corporation, Japan and; particle size 5 *μ*m). Samples (10 *μ*L) were injected via autosampler. The mobile phase consisted of *n*-hexane and isopropyl alcohol (90 : 10) and the flow rate was maintained at 1 mL·min^−1^. A DAD detector was used at a wavelength of 220 nm. Retention time of (S), (R)-1-phenyl-2-propyn-1-ol, and (R)-ester were 7.6 min, 6.5 min, and 4.4 min, respectively.

The enantioselectivity ratio (*E*) and conversion (*c*, %) were calculated from the enantiomeric excess of the substrate (ee_*s*_, %) and product (ee_*p*_, %) based on the following:

(1) $$\begin{array}{c} E=\frac{\ln\left[\left(1-c\right)\left(1-{\text{ee}}_{s}\right)\right]}{\ln\left[\left(1-c\right)\left(1+{\text{ee}}_{s}\right)\right]}, \end{array}$$

where

(2) $$\begin{array}{c} c=\frac{{\text{ee}}_{s}}{{\text{ee}}_{s}+{\text{ee}}_{p}},\\ {\text{ee}}_{s}=\frac{B_{\left(\text{S}\right)}-B_{\left(\text{R}\right)}}{B_{\left(\text{S}\right)}+B_{\left(\text{R}\right)}},\\ {\text{ee}}_{p}=\frac{Q_{\left(\text{S}\right)}-Q_{\left(\text{R}\right)}}{Q_{\left(\text{S}\right)}+Q_{\left(\text{R}\right)}}, \end{array}$$

where *B*_(R)_, *B*_(S)_, *Q*_(R)_, and *Q*_(S)_ denote area under the curve of (R)-1-phenyl-2-propyn-1-ol, (S)-1-phenyl-2-propyn-1-ol, and their corresponding esters, respectively.

## 3. Results and Discussion

### 3.1. Comparison of Microwave Irradiation and Conventional Heating

In order to compare the effect of microwave irradiation and conventional heating, the enzymatic resolution of (±)-1-phenyl-2-propyn-1-ol was performed in both modes using vinyl acetate as the model acyl donor and identical reaction conditions. It was observed that the reaction rate was increased about 1.28-fold from 1.16 × 10^−5^ mol·dm^−3^·s^−1^ to 1.49 × 10^−5^ mol·dm^−3^·s^−1^ under microwave irradiation. The *E* and ee_*s*_ under microwave irradiation increased by 1.61- and 1.27-fold, respectively. The final conversion of 48.78%, with *E* and ee_*s*_ of 334 and 93.25%, respectively, was obtained within 2 h under microwave irradiation. While, in conventional heating, a conversion of 42.85% with *E* and ee_*s*_ of 207 and 73.33%, respectively, was obtained. Under microwave irradiation, the ions or dipole of the reactants align in the applied electric field. As the applied field oscillates, the dipole or ion field tries to realign itself with the alternating electric field and, in the process, energy is released in the form of heat through molecule collisions and dielectric loss [24]. The quantity of heat released is directly related to the ability of the matrix to align itself with the frequency of the applied field. Microwave irradiation leads to efficient in situ heating, resulting in uniform distribution of temperature throughout the reaction mass. It is thus likely that minor conformational alterations in enzyme structure can also improve access of reactant to the active site, as compared to conventional heating [25]. A control experiment in the absence of enzyme did not show any conversion which indicated that there must be a definite synergism between enzyme catalysis and microwave irradiation. Thus, all further studies were carried out using microwave irradiation.

### 3.2. Effect of Different Catalysts

**Figure 1:**
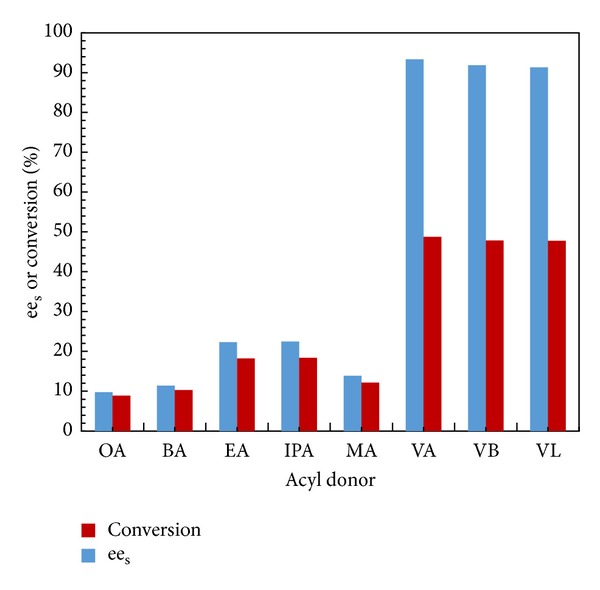
Effect of acyl donor. (Reaction condition: 1-phenyl-2-propyn-1-ol—0.001 mol; acyl donor—0.001 mol; *n*-hexane up to 15 cm^3^; speed of agitation—300 rpm; catalyst loading—10 mg; temperature—60°C, OA—Octyl acetate, BA—Butyl acetate, IPA—Isopropyl acetate, EA—Ethyl acetate, MA—Methyl acetate, VA—Vinyl acetate, VB—Vinyl butyrate, and VL—Vinyl laurate).

In order to compare the activities of three different commercially available immobilized lipases such as Novozym 435 (*Candida antarctica* lipase B), Lipozyme RMIM (*Rhizomucor miehei* lipase), and Lipozyme TLIM (*Thermomyces lanuginosus* lipase) for resolution of (±)-1-phenyl-2-propyn-1-ol under microwave irradiation, transesterification reaction was performed using vinyl acetate as the model acyl donor under similar conditions (Figure 1). It was observed that Novozym 435 showed highest activity among the different catalyst employed in this reaction. With Novozym 435, 48.78% conversion and 93.3% ee_*s*_ were obtained. While Lipozyme RMIM and Lipozyme TLIM gave conversions of only 6.37% and 4.4%, respectively. It is reported that both Lipozyme RM IM and Lipozyme TL IM are mainly used to interesterify bulk molecules such as fatty acid derivatives and they have less activity in the resolution of racemic molecules [26]. Hence, further study was carried out using Novozym 435 as the catalyst.

### 3.3. Effect of Acyl Donors

**Figure 2:**
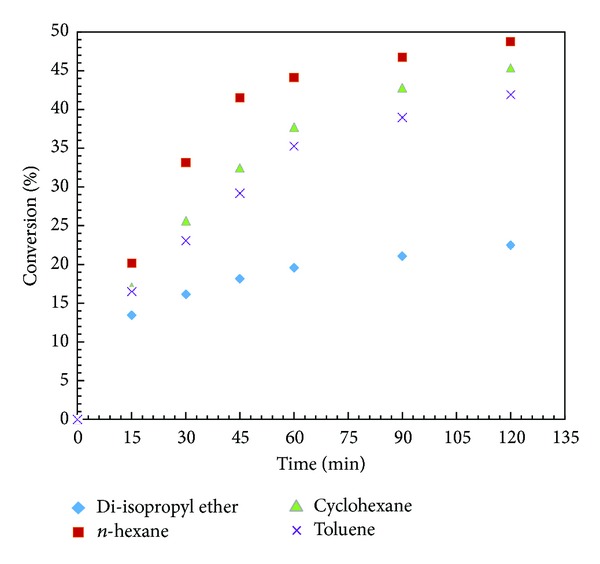
Effect of solvent. (Reaction condition: 1-phenyl-2-propyn-1-ol—0.001; vinyl acetate—0.001 mol; solvent up to 15 cm^3^; speed of agitation—300 rpm; catalyst loading—10 mg; temperature—60°C).

Acyl donors have the ability to change the enzyme activity and selectivity in nonaqueous medium [27, 28]. Effect of different acyl donors was studied by using 1 : 1 mole ratio of racemic alcohol to acyl donor, with 10 mg enzyme loading at 60°C in *n*-hexane as solvent for 2 h. The liquid phase volume was made up to 15 cm^3^ under microwave irradiation (Figure 2). The acyl donors were methyl acetate (MA), ethyl acetate (EA), isopropyl acetate (IPA), butyl acetate (BA), octyl acetate (OA), vinyl acetate (VA), vinyl butyrate (VB), and vinyl laurate (VL). It was observed that both enantioselectivity and conversion increased with increase in alcoholic carbon chain length of alkyl ester from methyl to isopropyl acetate, whereas further increase in alcoholic chain length from C4 to C8 led to decrease in conversion and enantioselectivity of reaction. As compared to alkyl esters, vinyl esters gave good conversion and enantioselectivity. Vinyl alcohol produced during reaction is instantaneously and irreversibly tautomerized to acetaldehyde, which escapes due to its low boiling point and thus there is no inhibition by the coproduct vinyl alcohol. Increasing the chain length of vinyl esters has not shown any significant differences in conversion and enantioselectivity of reaction. Maximum conversion (48.78%) and enantioselectivity (93.25%) were obtained with vinyl acetate vis-a-vis other vinyl esters. Thus, further studies were conducted by using vinyl acetate as the acyl donor.

### 3.4. Effect of Reaction Media

A proper selection of organic media is necessary for any enzymatic reaction. It has been well reported that organic solvents have the ability to alter the enzyme conformational structure that leads to changes in their activity and also regio-, and stereo-selectivity. Thus, proper selection of solvent is the most important parameter for lipase catalyzed reactions [29]. A number of experiments were performed to study the effect of solvents such as hexane (log⁡⁡*P* = 3.6), cyclohexane (log⁡⁡*P* = 3.2), toluene (log⁡⁡*P* = 2.5), and diisopropyl ether (log⁡⁡*P* = 2) under similar conditions (Figure 2). Highest conversion (48.78%) and enantiomeric excess (ee_*s*_ = 93.25%) were observed when *n*-hexane was employed as the solvent whereas low conversion (22.45%) and enantiomeric excess (ee_*s*_ = 28.96%) were observed in di-isopropyl ether, which has the lowest log⁡⁡*P* value among the solvents used in this study. On the contrary, conversions of 45.37% and 41.93% were obtained with cyclohexane and toluene, respectively. It has been reported that a tiny water layer around the enzyme particle is necessary to maintain its structure and activity. Solvents having high log⁡⁡*P* are hydrophobic in nature but do not strip the water layer around enzyme particle and its structure is not altered [30]. Thus, they show high enzyme activity. Further work was carried out using n-hexane as the solvent.

### 3.5. Effect of Speed of Agitation

**Figure 3:**
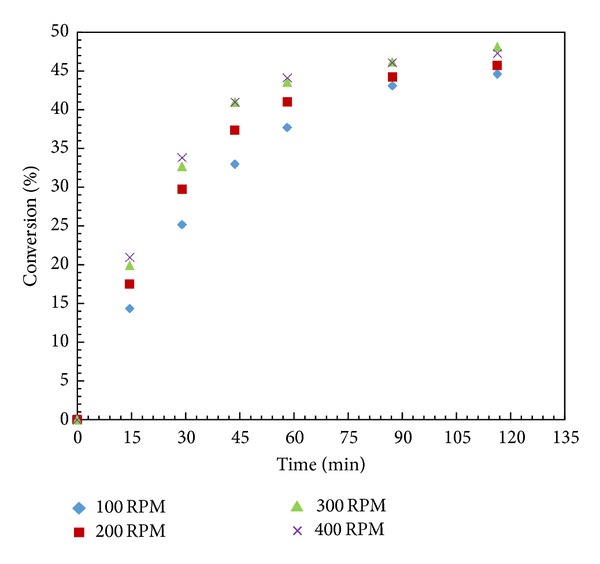
Effect of agitation speed. (Reaction condition: 1-phenyl-2-propyn-1-ol—0.001; vinyl acetate—0.001 mol; *n*-hexane up to 15 cm^3^; speed of agitation 100–400 rpm; catalyst loading—10 mg; temperature—60°C).

In immobilized enzyme catalyzed reactions, the reactants have to transport from bulk mixture into enzyme active site in the porous particle through convection and diffusion processes [31]. The magnitudes of external mass transfer resistance and intraparticle diffusion limitation play a crucial role in these processes. External mass transfer resistance can be overcome by choosing appropriate speed of agitation. A number of experiments were performed in the range of 100 to 400 rpm by taking 0.001 mol racemic alcohol and vinyl acetate each and 10 mg Novozym 435 made up to 15 cm^3^ with *n*-hexane at 60°C (Figure 3). Both the conversion and rate of reaction increased with an increase of agitation speed from 100 to 300 rpm. There was a marginal change in conversion and rate of reaction at 400 rpm. This indicated that there was no external mass transfer limitation above 300 rpm. The external mass transfer and intraparticle diffusion rates were further evaluated by comparing the time constants for reaction (*t*_*r*_) and diffusion (*t*_*d*_) using theoretical calculations. These are defined as follows: *t*_*r*_ = *C*_0_/*r*(*C*_0_) and *t*_*d*_ = *D*_S_/(*k*_SL_)^2^, if *t*_*r*_ ≫ *t*_*d*_ means that the reaction was not mass transfer controlled [32]. Both *C*_0_ and *r*(*C*_0_) were determined experimentally and their values were obtained as 0.06667 mol·dm^−3^ and 1.4916 × 10^−5^ mol·dm^−3^·s^−1^. Diffusivity of racemic alcohol at 60°C was calculated using Wilke-Chang equation as 5.1063 × 10^−5^ cm^2^ s^−1^ [33]. The average diameter of the support particle was taken as 0.06 cm since the particle size ranged between 0.03 and 0.09 cm. The value of mass transfer coefficient of liquid phase was calculated, by using Sherwood number of 2, to be 0.5301 cm·s^−1^. From these values, the calculated time constants for reaction and diffusion were 4.469 × 10^3^ s and 1.817 × 10^−4^ s. The time constant for the reaction was much higher than diffusion indicating that there was no external mass transfer resistance.

Further, it is necessary to rule out the intraparticle diffusion limitation. It could be done by comparing the rate of substrate diffusion per unit interfacial area (*k*_SL_ *C*_0_) with the reaction rate per unit area (*φr*_0_/*a*), where *φ* is the phase volume ratio and “*a*” is the interfacial area per unit volume of organic phase [32]. Assuming the particle was spherical, *φ*/*a* = *R*_*p*_/3, where *R*_*p*_ is the radius of the particle. It was found that the value of *k*_SL_ *C*_0_ = 0.0353 mol·cm^−2^·s^−1^ and *φr*_0_/*a* = 1.492 × 10^−7^ mol·cm^−2^·s^−1^. It indicated that the rate of substrate diffusion per unit interfacial area was much higher than the reaction rate per unit area. This suggested that there was no intraparticle diffusion limitation. Thus, the reaction was controlled by intrinsic enzyme kinetics. Therefore, further experiments were performed at a speed of 300 rpm.

### 3.6. Effect of Temperature

**Figure 4:**
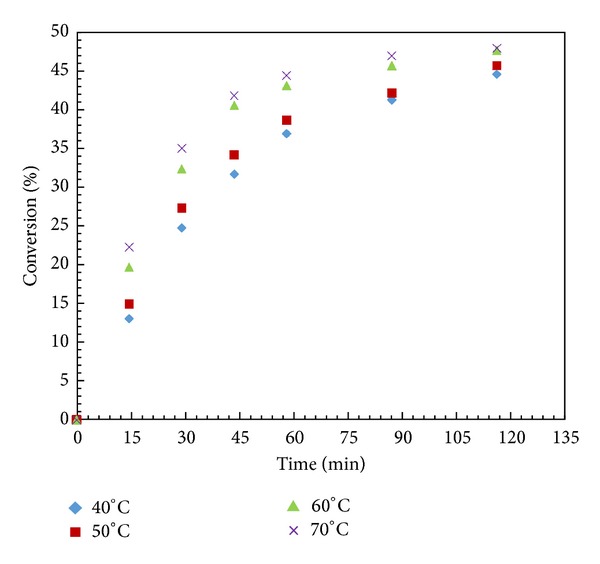
Effect of temperature. (Reaction condition: 1-phenyl-2-propyn-1-ol—0.001; vinyl acetate—0.001 mol; *n*-hexane up to 15 cm^3^; speed of agitation 300 rpm; catalyst loading—10 mg; temperature—40–70°C).

It has been reported in most of the literature that temperature plays a critical role in any enzyme catalyzed reaction. It will have an effect on the enzyme activity, enantioselectivity, and rate of reaction because temperature has pronounced effect on viscosity, solubility, and diffusivity of reactants and products. However, at high temperature, the changes in conformation of protein structure will affect their activity and selectivity [34]. Several experiments were performed in the range of 40 to 70°C under similar conditions (Figure 4). It was observed that both conversion and rate of reaction increased with increase in temperature up to 60°C beyond which there was no change in conversion. The enzyme particles may either get denatured or intraparticle diffusion resistance sets in. Further, Arrhenius plot was obtained by plotting the ln (initial rate) versus 1/*T* for both modes of heating and activation energies obtained for microwave and conventional heating were 17.39 kJ·mol^−1^ and 15.93 kJ·mol^−1^, respectively. These values are similar to those reported for most of the enzymatic reactions [35]. In the case of enantioselectivity, it was observed that the *E* value increases as temperature increased from 40 to 70°C. It has been reported that enantioselectivity is dependent on reaction temperature and is controlled by enthalpy and entropy of reaction [36]. The following equations were used to relate these terms:

(3) $$\begin{array}{c} \ln E=-\frac{\Delta_{R-S}\Delta H^{‡}}{R}\cdot \frac{1}{T}+\frac{\Delta_{R-S}\Delta S^{‡}}{R},\\ \Delta_{R-S}\Delta G^{‡}=-RT\ln\left(E\right). \end{array}$$

**Figure 5:**
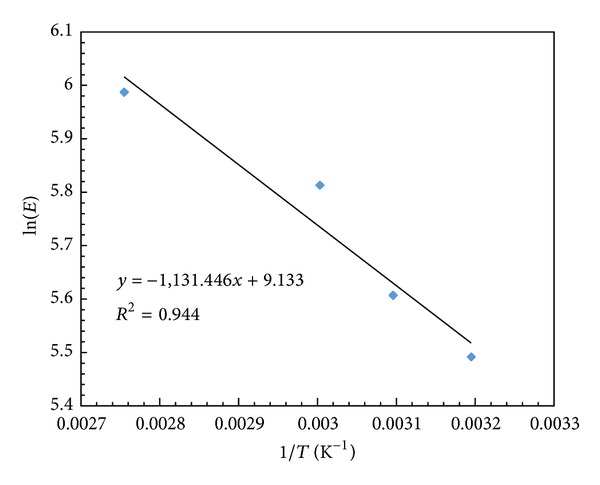
Graphical determination of Δ_*R*−*S*_Δ*H* ^‡^ and Δ_*R*−*S*_Δ*S* ^‡^.

The enthalpy and entropy values were calculated by plotting the ln⁡(*E*) values against the reciprocal reaction temperature (Figure 5). The values of enthalpy and entropy obtained were 9.406 kJ·mol^−1^ and 75.93 J·mol^−1^·K^−1^, respectively. The difference in transition state energy of both enantiomers (Δ_*R*−*S*_Δ*G* ^‡^) is −15.88 kJ mol^−1^. From these values, it was observed that the entropy value was much higher than enthalpy of system and it favors the maximum enantioselectivity at high temperature [37]. Since *E* value was found to be the highest at 60°C while maintaining a higher enzyme activity, 60°C was selected as the optimum temperature. Thus, further experiments were conducted at this temperature.

### 3.7. Effect of Enzyme Loading

**Figure 6:**
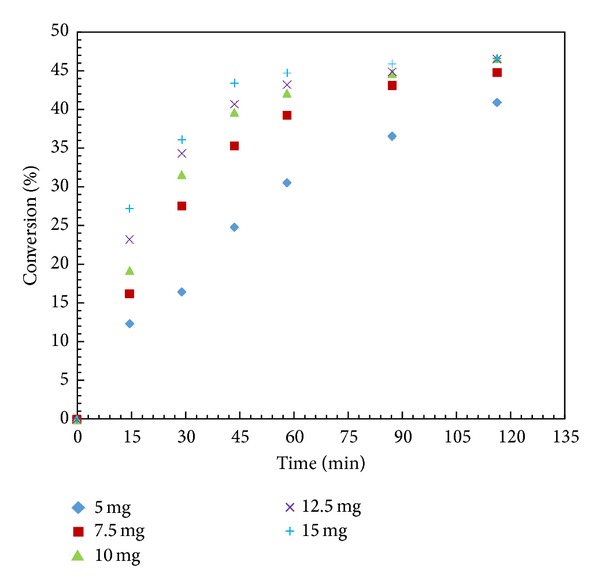
Effect of enzyme loading. (Reaction condition: 1-phenyl-2-propyn-1-ol—0.001; vinyl acetate—0.001 mol; *n*-hexane up to 15 cm^3^; speed of agitation 300 rpm; catalyst loading—5–15 mg; temperature—60°C).

To select the appropriate amount of enzyme loading, several experiments were performed in the range from 5 to 15 mg enzyme loading under similar conditions. The conversion and rate of reaction increased with increase in enzyme loading up to 10 mg; above this loading value, there were no substantial changes in conversion and rate (Figure 6). This would suggest that further addition of enzyme has no effect and it indicated that external mass transfer had limited the rate [29]. Further we observed that the initial rate increased linearly with enzyme loading up to 10 mg which clearly indicated that the reaction was kinetically controlled. Thus, further experiments were carried out at 10 mg as optimum enzyme loading.

### 3.8. Effect of Acyl Donor Concentration

**Figure 7:**
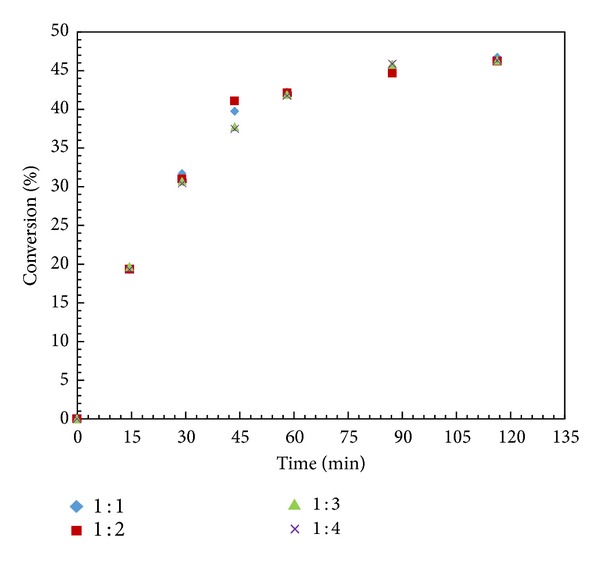
Effect of acyl donor concentration. (Reaction condition: 1-phenyl-2-propyn-1-ol—0.001; vinyl acetate—0.001–0.004 mol; n-hexane up to 15 cm^3^; speed of agitation 300 rpm; catalyst loading—10 mg; temperature—60°C).

A number of experiments were carried out to analyze the effect of acyl donor concentration on the rate and conversion in the resolution of (±)-1-phenyl-2-propyn-1-ol at 60°C with 10 mg of enzyme loading in n-hexane. The concentration of (±)-1-phenyl-2-propyn-1-ol was kept constant (0.001 mol), and vinyl acetate concentration varied from 0.001 to 0.004 mol; the mixture volume was kept constant at 15 cm^3^ by adjusting the amount of *n*-hexane addition. It was found that there were insignificant changes in conversion and rate of reaction with increase concentration of vinyl acetate (Figure 7). It might be possible that, at high concentration, the interaction between acyl donor and enzyme active site increases which leads to reversible competition with alcohol at the active site. Thus further reactions were conducted with 0.001 mole of vinyl acetate.

### 3.9. Catalyst Reusability

After completion of each run, the catalyst particles were filtered using a membrane filter. Multiple washes were given to the catalyst with fresh solvent (*n*-hexane), dried at room temperature for 12 h, and reused. It was found that there was a marginal decrease in conversion from 48.78 to 45.22% after three reuses, which was due to loss of enzyme during filtration (~2-3%) and drying. No make-up quantity was added. Thus, the enzyme was reusable. When the make-up quantity was added, almost the same conversion was obtained.

### 3.10. Kinetic Model

**Figure 8:**
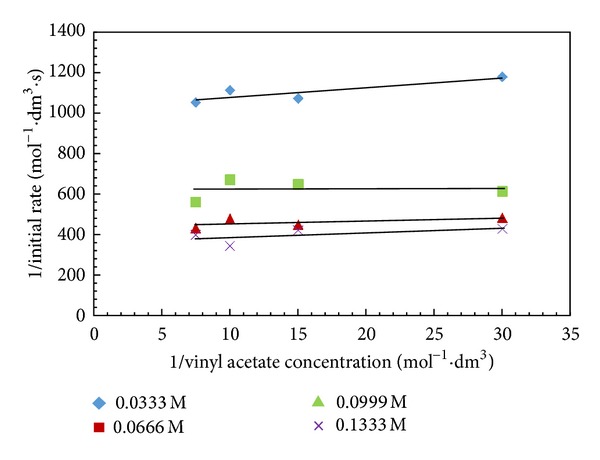
Lineweaver-Burk plot. (Reaction condition: 1-phenyl-2-propyn-1-ol—0.0333–0.1333 M; vinyl acetate—0.0333–0.1333 M; n-hexane up to 15 cm^3^; speed of agitation 300 rpm; catalyst loading—10 mg; temperature—60°C).

Several mechanisms have been proposed for lipase catalyzed reactions (e.g., ordered bi-bi mechanism, ping-pong bi-bi mechanism). However, ping-pong bi-bi mechanism was well adopted for most of the lipases catalyzed reactions. The first step of this mechanism is formation of an acyl-enzyme intermediate and the product is released between additions of substrates [38]. In the ordered bi-bi mechanism, the enzyme forms a ternary complex with both substrates and subsequently releases the products [26]. Thus, it was desirable to investigate the mechanism for enantioselective transesterification of (±)-1-phenyl-2-propyn-1-ol with vinyl acetate. For determination of initial rates, a number of experiments were carried out by changing the concentrations of both alcohol (0.0005–0.002 M) and vinyl acetate (0.0005–0.002 M) over a wide range under similar conditions. Initial rates were calculated systematically from the linear portion of the concentration-time profiles. The Lineweaver-Burk plots of reciprocal rate versus reciprocal concentration of vinyl acetate were made (Figure 8). From the plot, it is observed that there are no crossings of lines which rules out the possibility of ternary mechanism. At low concentrations of substrates, the slope of the lines is not influenced by the concentration of the fixed substrate. This is indicative of a mechanism that requires the dissociation of one product before the association of the second substrate to the enzyme-substrate complex. The curved shape at higher concentrations of 1-phenyl-2-propyn-1-ol can be used as an indication for formation of enzyme dead end complex with alcohol. The proposed mechanism is as follows:

(4) $$\begin{array}{c} E+A\overset{k_{1}}{\underset{k_{-1}}{⇄}}EA,\\ EA\overset{k_{2}}{\underset{k_{-2}}{⇄}}P+E^{\prime },\\ E^{\prime }+B\overset{k_{3}}{\underset{k_{-3}}{⇄}}E^{\prime }B,\\ E^{\prime }B\overset{k_{4}}{\underset{k_{-4}}{⇄}}EQ,\\ EQ\overset{\;K_{p}\;}{\to }Q+E. \end{array}$$

Inhibition steps by 1-phenyl-2-propyn-1-ol

(5) $$\begin{array}{c} E+B\overset{k_{i}}{\underset{}{⇄}}E_{i}B. \end{array}$$

From these observations, a ping-pong bi-bi mechanism with dead end alcohol inhibition was postulated. These assumptions are used to develop a reaction mechanism which is depicted in Cleland's notation, as shown in Figure 10.

By analogy to the classical mechanism of lipase catalysis, it is assumed that vinyl acetate (*A*) binds first to the free enzyme (*E*) and forms a noncovalent enzyme acetate complex (*EA*), which releases the first product, aldehyde (*P*) and (*E*′) modified enzyme. The second substrate alcohol (*B*) reacts with activated enzyme (*E*′) to give the complex (*E*′*B*) which gives the product ester (*Q*) and free enzyme (*E*). Along with this, alcohol (*B*) also forms the dead end complex (*E*_*i*_ *B*) by binding to free enzyme (*E*). Here, *B* is the R-isomer.

**Figure 9:**
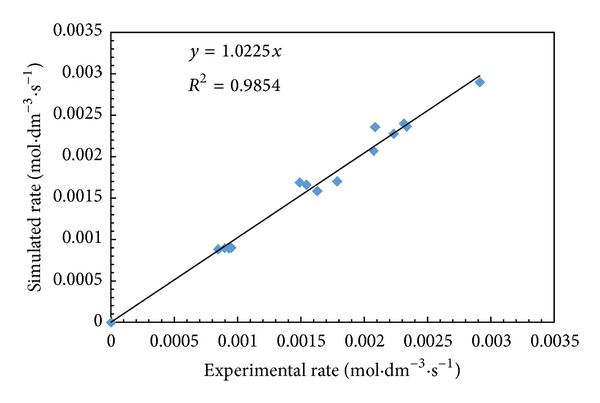
Parity plot of experimental versus simulated rates.

**Figure 10:**
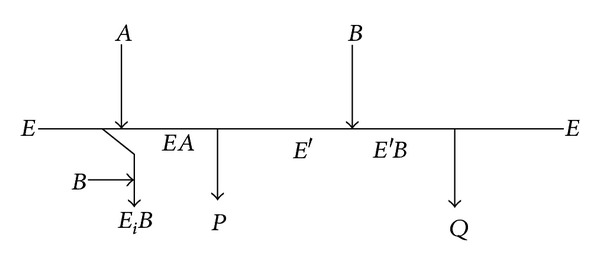


The kinetic representing this model for initial reaction rate can be expressed as

(6) $$\begin{array}{c} v=\frac{v_{m}\left[A\right]\left[B\right]}{K_{mB}\left[A\right]+K_{mA}\left[B\right]\left(1+\left(\left[B\right]/K_{i}\right)\right)+\left[A\right]\left[B\right]}, \end{array}$$

whereas *v* is the rate of reaction, *v*_*m*_ the maximum rate of reaction, [*A*] the initial concentration of vinyl acetate, [*B*] the initial concentration of (R)-1-phenyl-2-propyn-1-ol, and *K*_*mA*_ the Michaelis constant for vinyl acetate, as (S)-enantiomer of alcohol remains unreacted during the reaction, changes in concentration of (S)-enantiomer is neglected. *K*_*mB*_ the Michaelis constant for (R)-1-phenyl-2-propyn-1-ol, and *K*_*i*_ the inhibition constant for (R)-1-phenyl-2-propyn-1-ol. The initial rate data were used to determine the kinetic parameters of above mechanism by nonlinear regression analysis using the software package Polymath 5.1 (Table 1). The initial rates of reaction for different reactant concentration were simulated using above equation to verify the proposed kinetic model. The parity plot between simulated rate and experimental rate suggested that the proposed mechanism was valid for this reaction (Figure 9).

## 4. Conclusion

In this work, lipase catalyzed kinetic resolution of 1-phenyl-2-propyn-1-ol using vinyl acetate as acyl donor was studied under microwave irradiation. Among the employed catalysts, Novozym 435 was found to be the most effective catalyst in n-hexane as solvent. There was synergism between microwave irradiation and lipase. Both conversion and rate of reaction were increased under microwave as compared to conventional heating. Microwave irradiation leads to increase the affinity of the substrate toward the active site of lipase. Further, microwaves enhance the collision frequency of reactant molecules that leads to increase in entropy of the system. The effect of various parameters was studied on conversion and rate of reaction. The maximum conversion of 48.78% was obtained in 2 h using 10 mg enzyme loading with equimolar concentration of alcohol and ester at 60°C under microwave irradiation. From the progress curve analysis, it was found that the reaction followed the ping-pong bi-bi mechanism with dead end inhibition of alcohol. The kinetic parameters were refined by using nonlinear regression. There was a good fit between experimental and simulated rates. The preparation of chiral secondary alcohols using lipase catalyzed kinetic resolution is mild and clean as compared to chemical process.

## Figures and Tables

**Table 1:** Values of kinetic parameters for transesterification reaction.

Kinetic parameter	Value
*V*_*m*_ (mol·dm^−3^·s^−1^)	2.508 × 10^−2^
*K*_*mA*_ (mol·dm^−3^)	0.0019
*K*_*mB*_ (mol·dm^−3^)	0.8863
*K*_*i*_ (mol·dm^−3^)	0.00265

## Nomenclature

*A*: Initial concentration of vinyl acetate, mol·dm^−3^
*B*: Initial concentration of (R)-1-phenyl-2-propyn-1-ol, mol·dm^−3^
*E*: Free enzyme
*EA*: Enzyme-acyl complex with *A*
*E*′*B*: Modified enzyme-alcohol complex
*E*_*i*_*B*: Dead end enzyme-alcohol complex
*K*_*i*_: Inhibition constant for (R)-1-phenyl-2-propyn-1-ol, mol·dm^−3^
*K*_*mA*_: Michaelis constant for vinyl acetate, mol·dm^−3^
*K*_*mB*_: Michaelis constant for (R)-1-phenyl-2-propyn-1-ol, mol·dm^−3^
*P*: Acetaldehyde
*Q*: Ester
*v*: Rate of reaction, mol·dm^−3^·s^−1^
*v*_*m*_: Maximum rate of reaction, mol·dm^−3^·s^−1^
Δ_*R*−*S*_Δ*H*^‡^: Differential activation enthalpy
Δ_*R*−*S*_Δ*S*^‡^: Differential activation entropy
Δ_*R*−*S*_Δ*G*^‡^: Difference in transition state energy of both enantiomers
*T*: Temperature (K)
*R*: Gas constant (J·mol^−1^·K^−1^)
*E*: Enantiomeric ratio
ee_*s*_: Enantiomeric excess for substrate
ee_*p*_: Enantiomeric excess for product
*c*: Conversion.
